# Professional Quality of Life Factors and Relationships in Nursing and Psychiatric Nursing Students: An Exploratory Study

**DOI:** 10.1177/2377960821994394

**Published:** 2021-02-17

**Authors:** Kathryn M. Chachula

**Affiliations:** 1 College of Nursing, University of Saskatchewan, Saskatoon, Saskatchewan, Canada; 2 Department of Nursing, Faculty of Health Studies, Brandon University, Brandon, Manitoba, Canada

**Keywords:** nursing students, burnout, professional quality of life, self-efficacy, prior traumatic experience

## Abstract

**Introduction:**

Professional quality of life (ProQOL) that encompasses compassion satisfaction (CS) and compassion fatigue (CF) comprised of burnout (BO) and secondary traumatic stress (STS) has been raised as a world-wide issue for the nursing profession. Limited attention has been paid to the vulnerabilities of nursing students to ProQOL and the associated mechanisms.

**Purpose:**

Determine what factors are predictive of ProQOL in a population of undergraduate nursing and psychiatric nursing students. Methods: A cross-sectional survey was conducted comprised demographic questions and four validated measures: the Professional Quality of Life Scale (version 5), Core Self-Evaluations Scale, Perceived Stress Scale, and Life Events Checklist (version 5).

**Results:**

Students in long-term care-palliative care rotations reported significantly higher levels of BO in comparison to other care areas. Regression analysis revealed students with low self-efficacy and high perceived stress were predictive of BO. Students with increased exposures to prior traumatizing life events were predictive of STS. Students with high levels of self-efficacy and less intent-to-leave were predictive of having CS.

**Conclusion:**

Findings assist educators, clinicians, and policy makers in understanding at-risk clinical settings and predictors of ProQOL in pre-licensure students. Curricular recommendations that include mindfulness, coping and crisis peer-debriefing, and emotional intelligence are discussed.

## Introduction

This study investigated compassion satisfaction (CS) and compassion fatigue (CF) in pre-licensure students among nursing and psychiatric nursing programs at a Canadian university. Pre-licensure students are learners enrolled in a baccalaureate education program where upon completion, are eligible for licensing and registration under a health regulatory body. For example, on completion of a nursing education program, a graduate becomes eligible for professional licensure as a Registered Nurse (RN). CS refers to the level of reward a helper gains when carrying out ‘care’ or ‘help’ to others. In contrast, CF, comprised of secondary traumatic stress (STS) and burnout (BO), entails the negative aspects of work-related activities. Development of CF is highly concerning given that it is associated with “feelings of hopelessness and difficulties in dealing with work or in doing your job effectively” (Stamm, 2010, p. 17). This may impede provision of safe, competent, and ethical care that is in alignment with *Code of Ethics for Registered Nurses* (Canadian Nurses Association [CNA], 2017; Joinson, 1992).

Studies of nurses exposed to traumatic events revealed that a higher level of CS served as a protective factor against STS and BO (Hinderer et al., 2014). According to Michalec et al. (2013), limited attention has been paid to the vulnerabilities of nursing students to CF, the extent to which students may be suffering from BO, STS, and the associated mechanisms. Rudman and Gustavsson (2012) found that BO in undergraduate nursing students led to higher levels of intention-to-leave the nursing profession within one year upon workforce entry. A greater understanding is needed of the factors associated with CF in effort to prevent its formation and promote development of CS among nursing students prior to their entry into the workforce. Understanding what factors contribute to CF may assist nurse educators and researchers in formulating interventions and curricular planning strategies to support students and decrease the negative effects of caring for others.

## Background

### Compassion

Provision of compassionate care is a core value of professional nursing practice as highlighted in the *Code of Ethics for Registered Nurses* (Canadian Nurses Association [CNA], 2017). “Nurses engage in compassionate care through their speech and body language and through their efforts to understand and care about others’ health-care needs” (CNA, 2017, p. 8). In part, baccalaureate pre-licensure health curricula, serve to socialize students into their roles as care providers as students learn to foster and cultivate compassion. According to the American Association of Colleges of Nursing (AACN) and the Canadian Association of Schools of Nursing (CASN), a core essential of professional nursing education is the provision of competent, safe, ethical, and compassionate care delivery provided by the student nurse that is learned in baccalaureate education (AACN, 2008; CASN, 2015).

### Compassion Satisfaction (CS)

CS is defined as the positive aspects and pleasure a care provider gains despite any feelings of exhaustion and hardship (Stamm, 2002; Stamm, 2010). CS results from a transactional dynamic understood as the positive effects or ‘payments’ one gains as a result of caregiving, despite the ‘cost’ of helping others (Stamm, 2002). This is akin to Lazarus and Folkman’s (1984) theory of stress, appraisal, and coping where stress results from perceived imbalances between demands of a situation and the availability of resources to cope. According to this theory, the perception of one’s ability to cope has more importance than a particular stressor. The transactional nature of CS is evident in studies of nursing students who reported that CS is greater than CF (Mason & Nel, 2012; Mathias & Wentzel, 2017).

Feeling in control of a situation or stressor promotes coping and the perception that one has the resources to manage emotional distress. Stamm (2002) noted that only a fraction of individuals exposed to traumatic stressors developed symptoms associated with PTSD where gains in delivering compassionate care outweighs losses. Among nurses, there is often a sense of accomplishment in providing care to others that results in gaining rewards known as CS (Hinderer et al., 2014). Thus, CS acts as a protective factor against CF, and specifically STS (Hegney et al., 2014; Hinderer et al., 2014).

### Compassion Fatigue (CF)

According to Figley (1995), CF is the emotional pain caused in some care providers when exposed to a suffering individual. In a concept analysis of CF in nurses, Coetzee and Klopper (2010) reported that CF occurs when compassionate energy is not adequately restored. Carla Joinson (1992) was one of the first nurses to discuss CF in the published literature and referred to CF as being “emotionally devastating” requiring awareness to recognize when it is occurring. Joinson also acknowledged that the “outside sources that cause it are unavoidable” and that “‘caregivers’ personalities lead them towards it” (1992, p. 116). Joinson (1992) alluded that nurses may place high expectations upon themselves to provide care at an idealistic level and, when combined with other tasks such as paper work, care planning, delegation, and crisis management, these demands can leave the care provider depleted. For the purposes of the study, CF occurs when a care provider experiences greater STS and BO, rather than satisfaction, from care provision (Wijdenes et al., 2019). CF reflects the negative side of caring that diminishes the ability of a care provider to help others and is comprised of STS and BO.

### Secondary Traumatic Stress (STS)

STS results from the exposure of the care provider to the suffering of others who have or are experiencing stressful events (Boyle, 2011; Stamm, 2010). STS manifests in the care provider as feelings of fear, sleep difficulties, intrusive images, or avoiding reminders of traumatic experiences regarding the person for whom care was provided (Stamm, 2010). Figley (1995) acknowledged, “There is a cost to caring. Professionals who listen to clients’ stories of fear, pain, and suffering may feel similar fear, pain, and suffering because they care” (p. 1). While post-traumatic stress disorder (PTSD) arises due to primary trauma; STS arises due to empathetic hardship (Stamm, 2010). In consideration of pre-licensure health care students, approximately 40% of nursing students (Mathias & Wentzel, 2017) and midwifery students (Beaumont et al., 2016) are at risk of moderate levels of STS.

### Burnout (BO)

BO first arose in the literature in 1974 in a publication by Herbert Freudenberger which popularized the term (Freudenberger, 1974). Freudenberger described BO as a psychological, behavioural, and physical state that ranged from feelings of exhaustion and fatigue, frustration and anger, to physical manifestations (i.e., gastrointestinal illness); Freudenberger also noted that those who are committed to their work are at greatest risk of developing BO (Freudenberger, 1974). A concept analysis of CF in nursing revealed similar findings that included decreased energy, exhaustion, loss of power, physical complaints, irritability, intent-to-quit, and provision of poor-quality care (Coetzee & Klopper, 2010; Peters, 2018).

Similarly, Maslach and colleagues defined burnout as “a state of exhaustion in which one is cynical about the value of one’s occupation and doubtful of one’s ability to perform” (Maslach et al., 1996, p. 20). Where the Maslach Burnout Inventory (MBI) focused on exhaustion, cynicism, and professional efficacy, the Oldenburg Burnout Inventory (OLBI) focused on exhaustion and disengagement from work. Due to theoretical and psychometric concerns regarding a lack of theoretical depth, use of the MBI has reduced over time (Halbesleben & Demerouti, 2005), giving rise to alternate tools to assess for BO such the OLBI and ProQOL scales.

Within the current study, BO is defined as a component of CF where the care provider experiences decreased self-efficacy related to workload demands and increased perceived stress (Figley, 1995; Hegney et al., 2014; Rudman & Gustavsson, 2012; Stamm, 2010). Figley (1995) noted that BO has a gradual onset, which occurs as a result of STS, coupled with emotional exhaustion. Stamm (2010) characterized BO in care providers as feelings of being overwhelmed, unhappy, disconnected, and disengaged which occurs with a gradual onset. Moreover, BO is comprised of “exhaustion, frustration, anger and depression” related to a lack of a supportive work environment and increased workload demands (Stamm, 2010, p. 12). Figley (1995) attested that practitioners may endure feelings of deep sorrow and must understand their own limitations in alleviating pain suffered by clients who require help. Unfortunately, the factors that contribute to BO in nursing students are not well understood.

### Purpose

It is imperative that research be conducted about effective ways to support the future nursing workforce. Babenko-Mould and Laschinger (2014) acknowledged that nursing student placements in the clinical practice environment are positively or negatively influenced by the well-being of the workforce, which may lead to stress and BO formation that influence students’ career choice. Few studies have explored ProQOL variables within undergraduate nursing and psychiatric nursing programs. The aim of this study was to investigate the association of the ProQOL outcome variables comprised of CS, STS, and BO with intent-to-leave, measures of self-efficacy, perceived stress, and prior traumatizing events (PTEs). Exploring these phenomena may help nurse educators better understand the derivatives and associative factors of CS and CF, with the aim of supporting students while engaged in their undergraduate studies.

## Methods and Procedures

### Study Design

The study used an exploratory design that employed a cross-sectional, anonymous online survey. The survey was comprised of demographic questions and four validated measures in a population of students enrolled in the Bachelor of Science in Psychiatric nursing (BScPN) and Bachelor of Nursing (BN) programs within years two, three, and four at western Canadian university. Two key research questions guided the study. They were:

1. What are the inter-relationships between CS and the CF subscales of BO and STS?
2. What predictor variables (or factors) are associated CS, BO and STS in a population of pre-licensure students?

Several associations were anticipated between the predictor and outcome variables. As such, four hypotheses were generated related to the factors of interest. They were:

1. There will be a positive association between level of self-efficacy, a dimension of personality and emotional stability and CS.
2. There will be a positive association between perceived stress and STS.
3. There will be a positive association with PTEs and STS.
4. There will be a positive association between intention-to-leave and BO.

### Ethical Considerations

This study was approved by the Brandon University Research Ethics Committee and the University of Saskatchewan Behavioural Research Ethics Board. All participant information was anonymous and voluntary. Only aggregate data is reported to protect the identity of student respondents that adheres to ethical considerations for research involving human participants. A statement regarding informed consent was included in the title screen of the online survey.

### Study Population

Following receipt of ethical approval for the study, all full-time students enrolled in the BScPN and BN programs within years two, three, and four at a western Canadian university were invited to participate. Only full-time students were included in the study. Students on-leave from their program and those in their first general studies year were excluded from participating given that no clinical practica are embedded in the first year of each program.

### Sampling Procedure and Sample Size

Sampling occurred using a convenience sample of enrolled students within the BScPN and BN programs at one university institution. At the time of survey, there were 341 students eligible to participate. In the BN program there were 46 students in year two, 53 students in year three, and 45 students in year four. In the BScPN program, there were 81 students in year two, 53 in year three, and 63 in year four. An invitation to participate in the SurveyMonkey® online survey was issued through the University’s learning management system (i.e., Moodle). G*Power (Faul et al., 2007) software was used to determine the estimated sample size for linear multiple regression with five to seven predictor variables. The estimated sample size calculation was generated with alpha level at 0.05, effect size (*f* ^2^) set at medium or 0.15 (J. Cohen, 1988) and power at 0.8 to reduce the likelihood of a type II error (Polit & Beck, 2017). An estimated 92 to 103 participants were needed to obtain adequate statistical power to avoid a type II error.

### Instruments and Research Variables

#### The Professional Quality of Life Scale (ProQOL – Version 5)

The ProQOL tool is comprised of 30 items that are scored on a Likert scale ranging from 1 (never) to 5 (very often), with higher scores indicating higher levels on each subscale. The ProQOL Scale is comprised of three subscales with 10 items each that pertain to CS and CF comprised of BO and STS over the past four weeks. Reliability and validity of the tool have been demonstrated wherein the Cronbach’s alpha for CS was 0.88, burnout was 0.75, STS 0.81, and an overall alpha of 0.88 was obtained (Stamm, 2010). The ProQOL Scale offers a research measure with adequate convergent and discriminant validity, as well as construct validity when assessed using bifactor modelling of the three subscales (Geoffrion et al., 2019). In addition, the ProQOL Scale has been used in more than 200 peer reviewed papers (Stamm, 2010) involving studies of registered nurses, registered psychiatric nurses, and students of social work, midwifery, medicine, veterinary medicine, and nursing. In the current study, clarity was provided to participants that the terms ‘work’ and ‘job’ related to their role as a student in their program when providing care to patients, clients, and their families.

#### The Core Self-Evaluations Scale (CSES)

The CSES is a 12-item, self-report, Likert scale tool that pertains to self-efficacy (Judge et al., 2003). Each item is scored on a scale of 1 (strongly disagree) to 5 (strongly agree), with a total higher score being indicative of a person who is “well adjusted, positive, self-confident, efficacious, and believes in his or her own agency” (Judge et al., 2003, p. 304). Coefficient alpha reliability estimates reported by the tool creators were 0.84 (Judge et al., 2003, p. 316) with test-retest reliability at 0.81 (Judge et al., 2003). These findings indicate the tool is valid and reliable.

#### The Perceived Stress Scale (PSS)

The PSS is a 14-item, self-report, Likert scale tool (S. Cohen et al., 1983). Each item is scored on a scale of 0 (never) to 4 (very often) where a higher score indicates higher levels of perceived stress. The PSS provides a single summed score that assesses “the degree to which respondents found their lives unpredictable, uncontrollable, and overloading” within the last four weeks, influenced by the experience of “daily hassles, major events, and changes in coping resources” (S. Cohen et al., 1983, p. 387). Coefficient alpha reliability revealed scores of 0.84 to 0.86 were reported indicating the instrument is also a valid and reliable tool for use in undergraduate student populations (S. Cohen et al., 1983).

#### The Life Events Checklist (LEC – Version 5)

A modified version of the Life Events Checklist (LEC-version 5) was used to collect data regarding PTEs the participant has experienced (Weathers et al., 2018). The LEC is comprised of a 17 item, six-point, self-report tool that serves as “a screening measure of severity of trauma exposure” (F. Weathers, personal communication, October 30, 2018). The tool asks the participant to report if the stressful event indicated has: happened to the respondent, if they were exposed to the event as part of their job, if they witnessed the event occur to someone else, they learned about it happening to a family member or friend, if they are not sure it fits, or if it does not apply to them. Items that the respondent reports ‘happened to them’, ‘witnessed it’, or ‘exposed to it as part of the job’ were dummy-coded to receive a score of 1. All other responses received a score of 0. The tool is scored by determining the sum of individual questions (Jacobowitz et al., 2015). The LEC has demonstrated convergent validity with “adequate psychometric properties” when dichotomized to assess potentially traumatic event exposure with adequate test re-test reliability (Gray et al., 2004, p. 336).

An item was added to the above LEC tool adapted from the Life Stressor Checklist-Revised (LSC-R) developed by Wolfe et al. (1997). The added item read as: ‘Bothered, bullied, or harassed (i.e., jokes, verbal remarks) by someone in the work or school setting (i.e., another student, member of the health care team, manager, patient, or a teacher).’ The original LSC-R item 24 read as “Have you ever been bothered or harassed by sexual remarks, jokes, or demands for sexual favors by someone at work or school (for example, a coworker, a boss, a customer, another student, a teacher)?” (Wolfe et al., 1997, p. 7). The LSC-R has demonstrated an average kappa of 0.70 indicating adequate validity (Norris & Hamblen, 2004). This added item allowed the participant to report if they have experienced any negative behaviours that constitute bullying and harassment psychological stressors which have been noted in the nursing literature related to students (Budden et al., 2017).

Participants were also asked to report their intention-to-leave their program of study on a Likert scale item ranging from 1 (strongly disagree) to 5 (strongly agree). The item read as: ‘I think a lot about leaving the nursing/psychiatric program.’ The item was constructed based on studies published by Laschinger et al. (2012) and Rudman et al. (2014). The item provided an estimate of undergraduate students’ intentions of leaving their program. Laschinger et al. (2012) found that intention-to-leave was significantly correlated (*r* = .49, p < .05) with BO. Psychometric analysis of a similar question in a study by Rudman et al. (2014) revealed a Cronbach’s alpha of 0.75 indicating adequate validity. Rudman et al. (2014) argued that intention-to-leave may serve as a significant predictor of actually leaving the nursing profession.

#### Demographic Data

The demographic data collected in the study yielded descriptive data about the sample important for exploring statistical associations (Polit & Beck, 2017) with the previously identified variables. Demographic variables included age, relationship status, current clinical setting, average amount of sleep per night in a week, average amount of any hours of paid employment per week. Paid employment could include work related or unrelated to degree studies. If the student was not employed, the value zero was entered. Participants also indicated if they had a previous diagnosis of PTSD, anxiety, depression (yes or no) to control for confounding variables.

### Statistical Analysis

At the outset of the study, a data analysis plan was developed. This plan included using *Statistical Package for the Social Science*® (SPSS) for analysis of the variables. Scores were entered in keeping the guidelines for each of the tools to be adopted within the study, including how to treat missing values and reverse score items. Descriptive statistics and tests for normal distribution of the sample were conducted to allow for description of the sample and analysis of homogeneity, ensuring assumptions for parametric analysis were met for statistical testing.

## Results

Data were entered into SPSS version 26 and examined prior to analysis. Cases with missing data were explored and dependent variables were examined for homoscedasticity and normal distribution. Ninety-nine respondents accessed the survey, however, six were incomplete. The final sample size was adequate with 93/341 respondents resulting in a response rate of 27.27%. The average age of respondents was 24.6 years (SD = 5.27) and female (n = 89, 95.7%). Half of the respondents reported being single. There were 32 respondents (34.4%) from the BPN program and 61 (65.6%) from the BN program. Students in years two and three of their program were engaged in non-block clinical style with an alternating schedule of classes, labs, and clinical. In year four, all but four students were engaged in the final senior practicum structured in block clinical format with no other classes or coursework.

### Bivariate Inter-Correlation of Variables

Analysis of relationships among the dependent variables revealed that CS was inversely correlated with the CF subscales of BO (*r* = −.475, p < .001) and STS (*r* = −.164, p = .117). The inverse relationship between CS and STS did not reach statistical significance, however, the CF subscale measures of BO and STS were significantly positively correlated (*r* = .555, p < .001).

Analysis of relationships among the independent and dependent variables revealed a significant positive correlation between CS and the CSES, a measure of self-efficacy, *r* (91) = .370, p < .001. Secondly, there was a significant positive correlation between STS and the PSS *r* (91) = .455, p < .001. Thirdly, a significant positive correlation occurred between STS and PTEs measured by the LEC *r* (91) = .259, p < .05. Lastly, there was a significant positive correlation between BO and the intent-to-leave variable *r* (91) = .522, p < 0.001. To view a complete correlation matrix of the variables, refer to Table 1.

**Table 1.** Bivariate Correlation of Variables (n = 93).

Variable:	STS	CS	BO	PSS	CSES	LEC	PTSD	Depression	Anxiety	Age	Sleep	Hours worked	Intent to leave
STS	—												
CS	−.164	—											
BO	.555***	−.475***	—										
PSS	.455***	−.284**	.738***	—									
CSES	−.459***	.370***	−.744***	−.810***	—								
LEC	.259*	.123	.084	.165	−.109	—							
PTSD	.187	−.074	−.012	.214*	−.126	.137	—						
Depression	.183	−.113	.243*	.381***	−.359***	.208*	φ.386***	—					
Anxiety	.094	−.093	.179	.277**	−.199	.024	φ.271*	φ.462***	—				
Age	−.221*	.133	−.299**	−.233*	.304**	.201	−.077	.057	.040	—			
Sleep Hours	−.218*	−.135	−.251*	−.440***	.398***	−.041	.087	−.237*	−.183	−.006	—		
Hours worked	−.012	.031	−.075	−.085	.150	.080	−.045	−.174	.001	.149	.093	—	
Intent to leave	.356***	−.403***	.522***	.482***	−.537***	.003	.172	.155	−.023	−.200	−.111	−.055	—

*p<0.05; **p ≤ 0.01; ***p ≤ 0.001 (two-tailed testing); φ Phi Cross-tabs coefficient of dichotomous variables with Fisher’s Exact Test-.

### Clinical-Type Comparisons

**Figure 1.**
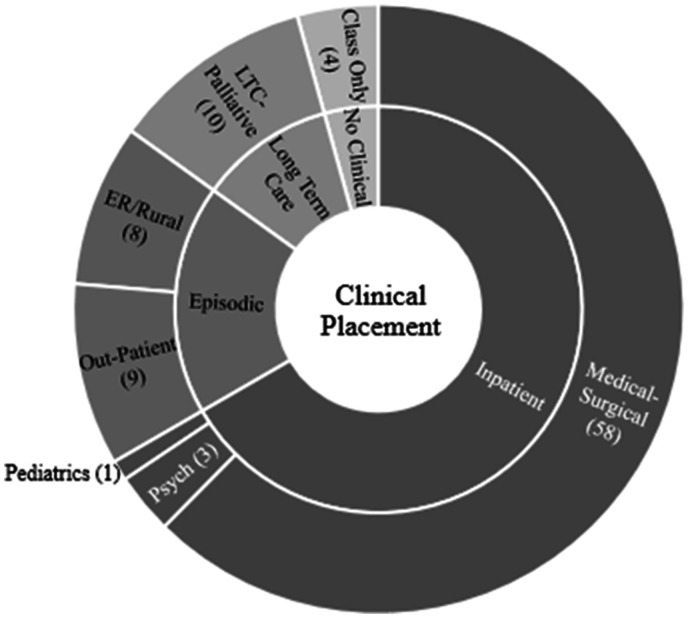
Student Clinical Setting Placements. Inpatient (n = 62); episodic care areas (n = 17); long-term care/palliative (n = 10); no clinical (n = 4).

Three categories of clinical placement sites were analysed using ANOVA testing. All in-patient units were clustered under one label named ‘Inpatient’ that included medical-surgical, paediatrics, acute psychiatry areas (n = 62). The second cluster was labelled ‘Episodic’ (n = 17) to reflect students in out-patient settings, and rural/emergency settings. The third cluster was comprised of students in long-term care (LTC)-palliative settings (n = 10). Students who were not in clinical (n = 4) were excluded from analysis. See Figure 1 for details. Levene’s statistic was not significant (p > .05) indicating limited variance among the re-clustered groups, therefore homogeneity of the sample was met.

Subsequently, a one-way ANOVA test with alpha set at 0.05 was completed for each of the dependent variables with Hochberg’s post-hoc analysis due to the unequal group sizes (Field, 2013; Hochberg, 1988). The findings for burnout revealed a significant difference (F_2,86_ = 3.695, MSE = 18.48, p = .029, 95% CI = 0.32–8.66) between the LTC-Palliative (M = 29.9, SD = 4.89) and Episodic care (M = 25.41, SD = 4.99) clinical placement areas. These findings showcase that students in the LTC-Palliative setting had significantly higher levels of BO. No statistically significant findings occurred for STS (F_2,86_ = 0.752, p = .474) and CS (F_2,86_ = 0.957, p = .388).

Comparisons among the independent variables with outcome variables was conducted prior to multiple hierarchal regression analysis. Analysis provided insight into student characteristics within the sample. Students with high levels of perceived stress were younger (*r* = −.233, p < .05), slept less (*r* = −.440, p ≤ .001), and had greater intention-to-leave their education program (*r* = .482, p ≤ .001). In contrast, students who reported high levels of self-efficacy had less perceived stress (*r* = −.810, p ≤ .001), were less likely to have a prior diagnosis of depression (*r* = −.359, p ≤ .001), were older (*r* = .304, p ≤ .01), slept more (*r* = .398, p ≤ .001), and had less intent-to-leave (*r* = −.537, p ≤ .001).

### Hierarchal Multiple Regression of Dependent Variables

Data were analysed using hierarchal multiple regression analysis (blockwise entry method in SPSS) of CS and the CF subscales (comprised of BO and STS). According to Field (2013), an effect size of 0.10 is considered small, 0.30 is medium, and 0.50 showcases a large effect. In accordance with the G*Power calculation five-to-six key variables were selected for entry into each model to limit a type II error. Variables were entered into the model informed by theory and hypothesis testing regarding the ProQOL Scale (Stamm, 2010). In addition, variables known from prior research published in peer-review literature were entered first, with new variables entered sequentially in accordance with hierarchal multiple regression (Field, 2013).

#### Burnout Regression Model

Homoscedasticity assumptions of the BO raw data for hierarchal multiple regression of the dependent variables were met. Theoretical and known factors related to BO from the published literature were entered into Model 1, 2, and 3 for hierarchal regression analysis (Table 2). Factors included age (Hegney et al., 2014), sleep hours (Stamm, 2010), depression (Harr et al., 2014), intent-to-leave the education program, perceived stress (C. D. Lin & Lin, 2016; Rudman et al., 2014), and core self-evaluation (Y. K. Lin et al., 2017). Model 1 and 2 findings indicated that the younger the student, the higher the BO level. The same principle occurred for sleep, in that lower amounts of sleep were predictive of burnout. A student who reported being diagnosed with depression also carried predictive capacity for burnout. Model 3 allowed the researcher to test if perceived stress, core self-evaluation, and intent-to-leave made a difference in predicting BO, over-and-above the previously entered variables in Models 1 and 2. The findings revealed perceived stress and core self-evaluation were significant contributors adding explanatory power of the model.

**Table 2.** Standardized Coefficients (Beta) for Hierarchal Regression Analysis of BO.

Variables	Model 1	Model 2	Model 3
Age	−.299**	−.313**	−.062
Sleep hours		−.202*	.083
Depression		.213*	−.045
Perceived Stress Scale			.418***
Core Self-Evaluations Scale			−.368**
Intent to Leave Education Program			.126
R^2^ Change	0.09	0.11	0.44
R^2^	0.090	0.196	0.635

*p < 0.05, **p ≤ 0.01, ***p ≤ 0.001.

Model 3: Effect size calculated in G*Power = 1.7397, Power = 1.000.

The final model predicting BO included age, sleep hours, depression, perceived stress, core self-evaluation, and intent-to-leave the education program. As predictors were entered into the three models, the R^2^ Change value increased sequentially. The model explained 63.5% of the variance in scores (F_6,86_ = 24.911, p ≤ .001) fully powered with a very large effect size of 1.74. Within the third model, core self-evaluation (*β* = −0.368, p ≤ .01) and perceived stress (*β* = 0.418, p ≤ .001) were significantly predictive of burnout. Greater levels of student self-efficacy measured by the CSES, was inversely related to burnout; whereas, perceived stress was significantly, related to greater levels of burnout as part of CF.

#### STS Regression Model

Following log 10 transformation of the STS dependent variable, the assumptions for regression analysis testing were met (Kolmogorov-Smirnov, p = .200; Shapiro-Wilk, p = .423). Subsequently, known factors of STS discussed in the nursing and allied health literature as well as theoretical variables of interest were entered into three models (Table 3). The predictors included age (Knight, 2010), average amount of sleep per night in a week (Stamm, 2010), PTEs measured by the Life Events Checklist (Jacobowitz et al., 2015; Stamm, 2010), followed by perceived stress (Harr et al., 2014; Paspaliaris & Hicks, 2010), intent-to-leave (Coetzee & Klopper, 2010; C. D. Lin & Lin, 2016; Peters, 2018; Rudman & Gustavsson, 2012), and core self-evaluation (Y. K. Lin et al., 2017; Mason, 2018).

**Table 3.** Standardized Coefficients (Beta) for Hierarchal Regression Analysis of STS.

Variables	Model 1	Model 2	Model 3
Age	−.234*	−.294**	−.162
Sleep hours		−.215*	−.069
LEC		.294**	.232*
Perceived Stress Scale			.129
Core Self-Evaluations Scale			−.178
Intent to Leave Education Program			.175
R^2^ change	0.06	0.13	0.13
R^2^	0.055	0.189	0.320

*p < 0.05, **p ≤ 0.01.

Model 3: Effect size calculated in G*Power = 0.4706, Power = 0.999.

As values were entered into the STS model, the R^2^ Change value increased from Model 1 and 2 and did not further increase from Model 2 to Model 3. This indicated that inclusion of the new predictors did not greatly contribute to explaining the overall variance. The final model explained 32% of the variance for STS (F_6,86_ = 6.756, p ≤ .001), powered at 99% with a moderate-to-large effect size of 0.47. The significant predictor of STS within Model 3 was PTEs (*β* = 0.232, p < 0.05). All other variables were not unique, significant predictors of the STS subscale.

#### CS Regression Model

Following log 10 transformation of the CS dependent variable, assumptions for normal distribution were met (Kolmogorov-Smirnov, p = .191; Shapiro-Wilk, p = .199). Subsequently, factors of interest informed by the published literature for CS were entered into Model 1, 2, and 3 for hierarchal multiple regression analysis (Table 4). Predictors included age (Hegney et al., 2014), average amount of hours of sleep per night in one week (Stamm, 2010), core self-evaluation (Mason, 2018), perceived stress, and intent-to-leave the education program (Coetzee & Klopper, 2010; Harr et al., 2014; Paspaliaris & Hicks, 2010; Peters, 2018; Rudman & Gustavsson, 2012; Rudman et al., 2014). As the five predictive variables were entered into each model, the R^2^ Change value increased from Model 1 and 2, and subsequently decreased in Model 3 as perceived stress and intent-to-leave were added.

**Table 4.** Standardized Coefficients (Beta) for Hierarchal Regression Analysis of CS.

Variables	Model 1	Model 2	Model 3
Age	.120	−.037	−.043
Sleep hours		−.332**	−.305**
Core Self-Evaluations Scale		.509***	.339*
Perceived Stress Scale			−.026
Intent to Leave Education Program			−.261*
R^2^ change	0.014	0.210	0.049
R^2^	0.014	0.225	0.273

*p < 0.05, **p ≤ 0.01, ***p ≤ 0.001.

Model 3: Effect size calculated in G*Power = 0.3755, Power = 0.998.

The final model for CS explained 27% of the variance for CS (F_5,87_ = 6.546, p ≤ .001), powered at 99.8% with a moderate effect size of 0.376. Age and levels of perceived stress were not significant predictors of CS. Hours of sleep were inversely associated with CS (*β* = −0.305, p ≤ .01). Students with greater self-efficacy measured by the CSES was a significant predictor of CS (*β* = −0.339, p <.05); in addition, those who reported less intent-to-leave was predictive of CS (*β* = −0.261, p <.05).

## Discussion

### Dependent Variable Inter-Relationships

Inverse relationships of CS with CF measures of STS and BO were consistent with the published literature in undergraduate pre-licensure students (Flinton et al., 2018; Mason & Nel, 2012). Although the inverse relationship of CS and STS was not statistically significant in the current study; these findings were akin to those by McArthur et al. (2017) in a sample of Australian veterinary medicine students. Furthermore, the direction of the relationships among the dependent variables is consistent with the transactional nature of stress and coping (Lazarus & Folkman, 1984) and Stamm’s theoretical model of CS and CF (Stamm, 2010). Cronbach’s alpha scores achieved in the study were satisfactory with CS and STS achieving scores greater than 0.70. The BO alpha was 0.67, however, this is higher than the BO alpha of 0.48 achieved by Michalec et al. (2013) in their study of American nursing students.

### Clinical Settings and Dependent Variables

Students placed in LTC-palliative care settings had statistically significant higher levels of burnout as opposed to episodic care areas and acute in-patient units. According to Potter et al. (2010) nurses who work in home health settings and oncology clinics were at greater risk of CF. Berger et al. (2015) reported that feelings of exhaustion and hardship with subsequent CF were present in nurses involved in end-of-life situations. Moreover, long-term care settings breed the ‘perfect storm’ of clients who have numerous comorbidities, complex and demanding care needs, and often poor patient-to-nurse staff ratios combined with the rising tide of horizontal/lateral and vertical workplace violence (Littlejohn, 2012). Students in this type of clinical environment must also contend with the additional challenge of meeting course and clinical objectives, which may be an additional source of strain and exhaustion giving rise to BO.

### Predictor and Dependent Variables

#### Burnout

BO is one component of CF within the ProQOL Scale that was analysed using regression analysis. The finding that high levels of perceived stress and low self-efficacy were predictive of BO were consistent with those found by Mason (2018) in that CF is closely related to nursing students with pessimistic attitudes. This negative or pessimistic perspective is reflective of those who scored lower on the core self-evaluation measure. These findings support that negative affectivity or neuroticism are significant predictors of BO (Flinton et al., 2018; Škodová et al., 2013). Among university students with Type D Personality (prone to negative emotion or a pessimistic affect), Williams and Wingate (2012) found that social support and emotion-focused coping can decrease perceived stress. In a study of nurses and nursing students, Garrosa et al. (2008) found that significant predictors of BO included job stressors such as workload, experience with pain and death, conflict interactions, younger age, and role ambiguity.

Within the current study, being married/partnered or single, having a prior diagnosis of anxiety, and work hours were not significantly associated with CS or CF. Dasan et al. (2015) found that being single was associated with BO where being married offers some protective effect. McArthur et al. (2017) found, in part, that female veterinary students who had paid employment in a veterinary clinic unrelated to a school placement had higher amounts of CS. In contrast, Harr et al. (2014) in their study of master and bachelor of social work students found that the number of hours employed and BO were statistically significant. These conflicting findings imply that further research is needed.

#### Secondary Traumatic Stress

STS is the second component of CF measured within the ProQOL Scale. As indicated by the STS hierarchal regression model, having a higher number of PTEs was predictive of STS. This finding is further supported by Stamm’s (2010) theoretical model of CS and CF that incorporated primary traumatic exposures as playing a role in development of STS. In a study of nursing students, researchers found that the more adverse childhood experiences (ACEs) female students had experienced, the higher the levels BO and depression when compared to men (McKee-Lopez et al., 2019). These findings imply that students entering the nursing and psychiatric nursing profession with numerous PTEs may be at significant risk to STS. These students may require additional counselling supports during their education to develop positive coping strategies prior to entering the workforce as registered practitioners. Students should be encouraged to mobilize personal coping supports and access counselling services available at the university and/or through private counselling services often covered by student union dues or under other health plans.

#### Compassion Satisfaction

Despite having less sleep, students with high levels of self-efficacy reported less intent-to-leave and higher levels of CS. Of note, the current study did not explore *quality* of sleep in conjunction with *quantity* of sleep highlighting an area of future study. Salmela-Aro et al. (2009) found that undergraduate students with high levels of optimism had high levels of work engagement with low levels of BO up to 17 years post-graduation. According to Garrosa et al. (2008), students with a hardy personality who felt they had a greater sense of control and commitment were inversely associated with BO. These findings support that high levels of self-efficacy protect students from BO and potentiate CS formation. Of note, positive sleep hygiene practices improve academic performance and neurocognitive performance indicating that educators should discuss the importance of sleep for academic success (Abdulghani et al., 2012; Gilbert & Weaver, 2010). More research is needed to explore the link between sleep and CS.

#### Limitations

To date, this is the only study that has explored outcome variables of ProQOL within a sample of BScPN and BN students in Canada. Four key limitations are noted within the study that includes use of convenience sampling, limited generalizability, self-report screening tools, and timing of the survey. Participants were recruited from a convenience sample of currently enrolled full-time students at a single university. Only 27% of the eligible student population participated. Although a typical response rate of approximately 30% in education research is the norm, the non-randomness, small sample size, and homogeneity of the sample introduces sampling bias that limits generalization of findings (Polit & Beck, 2017). Use of self-report screening tools and questionnaires introduce bias if responses are inaccurately reported, the reliability of the measures decrease and measurement error occurs (Polit & Beck, 2017). Many of the tools, including the ProQOL Scale adopted within the study, serve as screening tools. While student nurses may not be considered professionals, it is important to note that students are socialized into the professional role of the nurse with expectations to adhere to values and competencies expected of registered, practicing nurses identified within the *Code of Ethics* (CNA, 2017). Similarly, “The Life Events Checklist is only intended to be a screening measure to evaluate exposure to possible Criterion A events…. It is very challenging to measure total trauma load” (F. Weathers, personal communication, October 30, 2018). The PSS continues to offer a valid, reliable, and empirically tested avenue to assess stress within university students and working adults. The survey was administered during the months of February and March 2020 prior to the novel coronavirus pandemic with subsequent closure of university settings.

### Implications for Practice

This study adds to a growing call for undergraduate pre-licensure programs to integrate measures that bolster students’ coping and self-efficacy during times of distress. The study findings revealed that students entering the BN and BScPN programs may not be prepared to face the stressors encountered during care provision. Curricular strategies that include a four-week mindfulness course, coping and crisis peer-debriefing workshops, and incorporating emotional intelligence development throughout pre-licensure curricula are potential strategies to address high levels of BO and STS. Fostering student self-care in pre-licensure programs is an essential measure to promote CS prior to entry into the workforce. Teaching and advocating that students be engaged in positive coping and self-care practices to sustain student well-being while enrolled in nursing and psychiatric nursing programs are warranted for a long-lasting career.

### Mindfulness

Development of mindfulness skills may serve to protect students against CF and promote the protective effects of CS (Clarkson et al., 2019). A four-week course that addresses weekly learning objectives centered on mindfulness can be integrated early within pre-licensure programming. Ouliaris (2019) promoted mindfulness meditation and reflective writing as two methods that can be integrated into curricula to increase student capacity for self-awareness. Chung et al. (2018) recommended four components of a mindfulness-based curriculum that involved: (1) One weekly 60-minute classroom session every week for four weeks; (2) Prerequisite reading assignments to accompany the classroom sessions with topics such as foundational wellness and mindfulness concepts, stress, burnout, and healthy practices; (3) Individual meditation practice and journal assignments; and (4) Developing a personalized wellness plan. Rees et al. (2016) advocated that students are taught mindfulness skills to prevent burnout, especially for students with high levels of neuroticism and maladaptive coping.

### Coping and Crisis Peer-Debriefing

Davies and Coldridge (2015) advocated that students should be trained in how to cope with traumatic situations. Coping and crisis peer-debriefing workshops can be integrated into curricula aligned with entry into clinical practice within their program. A student-drafted personal wellness plan assignment (Chung et al., 2018) embedded as part of a theory or clinical course may assist students in mobilizing their own coping supports during times of real and perceived crisis. In addition, crisis peer-debriefing education may serve as a benefit for students if a classmate is not comfortable seeking assistance from an instructor or if immediate counseling is not available. Grief training may also serve pre-licensure health students when efforts to save a life are unsuccessful or when providing care to clients faced by life threatening circumstances (Sikstrom et al., 2019).

### Emotional Intelligence

Interventions that foster emotional intelligence (Goleman & Boyatzis, 2017), civility, and positive student coping resources prior to their entry into the workforce are warranted. Nurturing emotional intelligence in students can serve to reduce stressors, mitigate workplace bullying, unfriendliness, and hazing within nursing (Littlejohn, 2012). Nurse educators and managers play a significant role in creating and leading environments that promote teamwork, positive working relationships, and positive working conditions (Hunsaker et al., 2015). Positive faculty role-modeling and curricula that fosters a culture centered on civility (Clark, 2017) that addresses bullying and workplace violence are advisable. These efforts can serve to promote provider resiliency, emotional regulation, and encouragement that enables flourishing.

Creating a positive teaching and learning environment that fosters openness, creativity, efficiency, organization, a sense of accomplishment, and overall positive psychology may play a role decreasing CF within student populations prior to their entry into the workforce. The *PERMA Model* (Seligman, 2011) comprised of *P*ositive emotion, *E*ngagement, *R*elationships, *M*eaning, and *A*ccomplishments provides educators an avenue to incorporate aspects of positive psychology within preparational curricula (Slavin et al., 2012). Integrating emotional intelligence development as part of an ‘emotional curriculum’ (van Zyl & Noonan, 2018) could serve as a proactive approach in developing student resilience, coping, self-management, social intelligence, leadership, and emotional self-awareness as learners and entry-level practitioners.

## Conclusion

The findings in this study highlight that pre-licensure nursing and psychiatric nursing students are not immune to low levels of CS and high levels of BO and STS that comprise CF within the ProQOL scale. Students in LTC-palliative care rotations reported significantly higher levels of BO in comparison to students placed on in-patient units such as medical-surgical areas and episodic care areas that included out-patient and emergency department settings. Regression analysis revealed that students with low self-efficacy and high perceived stress were predictive of BO. Students with increased exposures to PTEs were predictive of STS. Students with high levels of self-efficacy, less sleep, and commitment to their program with less intent-to-leave were predictive of having CS. Findings of the study assist educators, clinicians, and policy makers to better understand at-risk students and their associated clinical settings, as well as predictors of CS and CF in undergraduate nursing and psychiatric nursing students prior to entering the workforce as newly-licensed professionals. Curricular strategies that bolster students’ resilience, coping, and self-efficacy during times of stress, distress, and feelings of exhaustion are warranted prior to entry into the workforce.
